# [Corrigendum] Leonurine protects cardiac function following acute myocardial infarction through anti‑apoptosis by the PI3K/AKT/GSK3β signaling pathway

**DOI:** 10.3892/mmr.2023.12952

**Published:** 2023-02-02

**Authors:** Lin Xu, Xuejun Jiang, Fang Wei, Hongling Zhu

Mol Med Rep 18: 1582–1590, 2018; DOI: 10.3892/mmr.2018.9084

Subsequently to the publication of the above article, the authors have realized that Fig. 3 was published with an error: Essentially, the image selected for Masson's staining of the Leonurine group in Fig. 3A (the right-hand panel) was inadvertently selected from Fig. 4 in an article published previously by the same group [Zhu H, Jiang X, Li X, Hu M, Wan W, Wen Y, He Y and Zheng X: Intramyocardial delivery of VEGF165 via a novel biodegradable hydrogel induces angiogenesis and improves cardiac function after rat myocardial infarction. Heart Vessels 31: 963–975, 2016]. However, owing to the time that has elapsed since this article was published, the authors no longer had access to their original data; therefore, they were granted permission by the Editor to repeat the experiments shown in Fig. 3, and the revised version of Fig. 3 is shown below.

Note that this error did not significantly affect the results or the conclusions reported in this paper. All the authors agree to the publication of this Corrigendum, are grateful to the Editor of *Molecular Medicine Reports* for allowing them the opportunity to correct this error, and apologize to the readership for any inconvenience caused.

## Figures and Tables

**Figure 3.**
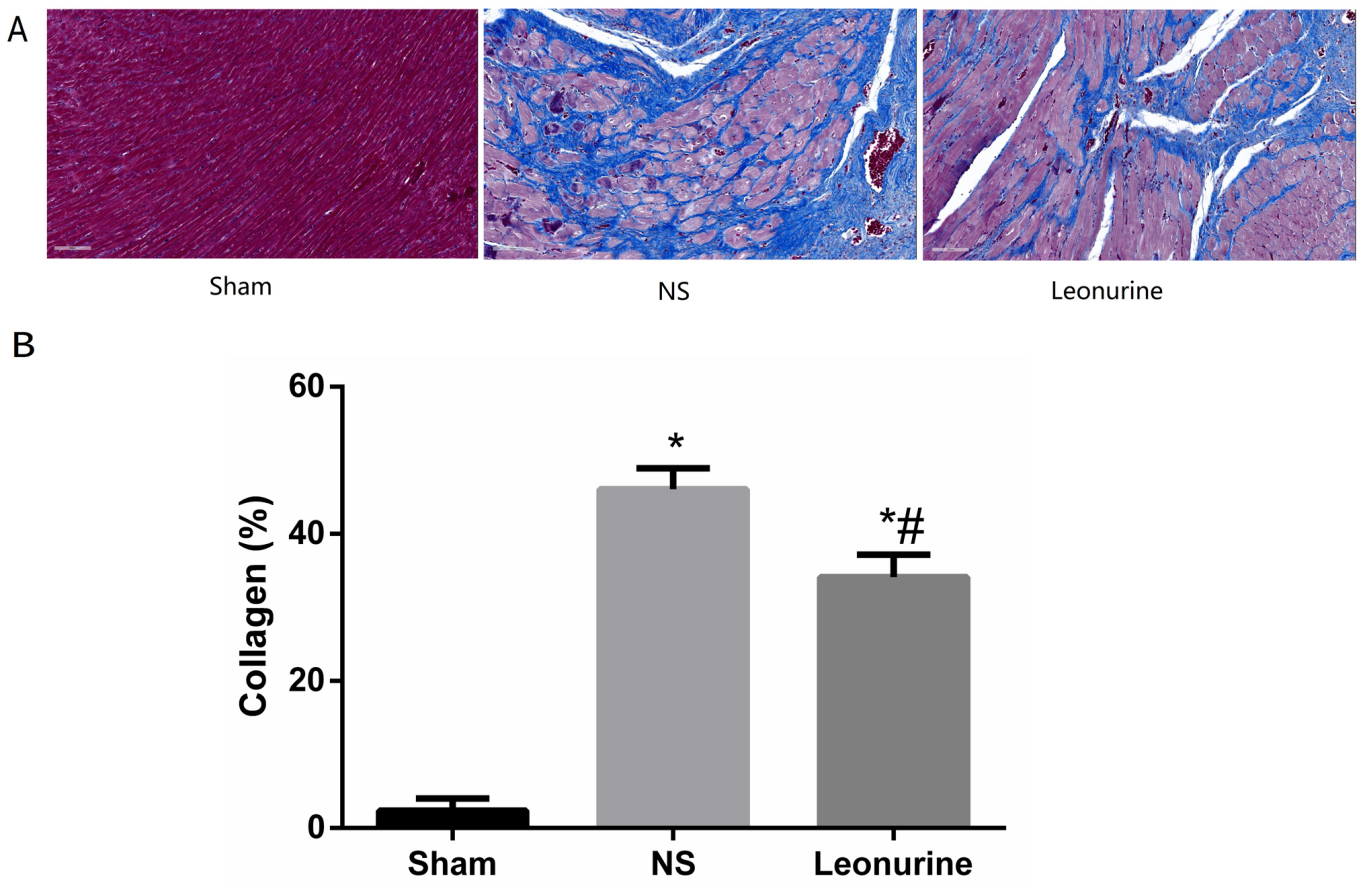
Collagen content 28 days after myocardial infarction. (A) Representative pictures of left ventricles from each group after Masson's Trichrome staining (magnification, ×200). (B) Collagen content as percentages at 28 days. Collagen content is calculated from the ratio of the collagen area to the area of the entire high-power field. The data are expressed as the mean ± standard deviation. *P<0.05 vs. sham, ^#^P<0.05 vs. NS. n=6 in each group. NS, saline group.

